# Awareness of the Malaria Vaccine in India

**DOI:** 10.7759/cureus.29210

**Published:** 2022-09-15

**Authors:** Chinar Singhal, Taiwo O Aremu, Pranjal Garg, Kunal Shah, Olihe N Okoro

**Affiliations:** 1 Department of Public Health, University of Minnesota, Minneapolis, USA; 2 Division of Environmental Health Sciences, School of Public Health, University of Minnesota, Minneapolis, USA; 3 Department of Pediatrics, University of Minnesota Medical School, Minneapolis, USA; 4 Department of Pharmaceutical Care and Health Systems, College of Pharmacy, University of Minnesota, Minneapolis, USA; 5 Department of Medicine, All India Institute of Medical Sciences, Rishikesh, IND; 6 Department of Medicine, Hinduhridaysamrat Balasaheb Thackeray Medical College, Mumbai, IND; 7 Department of Social and Administrative Pharmacy, University of Minnesota, Duluth, USA

**Keywords:** india, awareness, acceptability, malaria vaccine, malaria

## Abstract

Background: Malaria remains a serious public health problem in India. According to the World Health Organization (WHO), as per the 2021 report, India accounts for 83% of malaria cases in Southeast Asia. Various interventions have been implemented to control malaria's burden in India. In October 2021, the WHO approved the RTS,S/AS01 (RTS,S) malaria vaccine for administration in four scheduled doses in children five months of age to reduce the burden and severity of malaria. The objectives of this study were to assess public awareness about the vaccine among residents of India and determine any associations with demographic characteristics.

Methods: The study was a web-based, cross-sectional survey. The survey questionnaire was sent out electronically using Qualtrics® (Provo, UT) and remained active for 12 weeks (December 2021 to March 2022). The questionnaire was self-administered anonymously, using a link that was shared with people across India through social media platforms. A total of 2,371 respondents above 18 years of age and current residents of India participated in the study. The chi-square test was used to examine the association between awareness about the vaccine and demographic characteristics. A p-value of <0.05 was used to describe a statistically significant association.

Results: Most participants (71.95%) had heard about the malaria vaccine, and 68.75% favored making it a required childhood vaccine. Similarly, 67.27% indicated that they would encourage caregivers to get their children/wards vaccinated. Age, gender, educational status, residence, and caregiver status were associated with the awareness regarding the malaria vaccine (p < 0.05). Males, participants between 18 and 24 years old, and caregivers of children aged five years or less were more likely to be aware of the vaccine. Participants with higher education and residing in urban localities had more awareness of the vaccine.

Conclusion: The malaria vaccine has the potential to eradicate malaria in India, especially if included in the immunization schedule for children. However, it is critical that health policymakers target populations that are less aware of information on any intended rollout of the malaria vaccine to ensure rapid uptake toward the goal of eliminating malaria from India.

## Introduction

Malaria is a potentially fatal vector-borne disease caused by the *Plasmodium* parasite and contributes to higher rates of mortality and morbidity in India. As per the World Health Organization (WHO) World Malaria Report 2021, there were approximately 241 million cases reported across 85 different countries across the world [1]. This number has increased since 2019 (227 million cases). The number of cases per 1,000 population at risk (case incidence of malaria) was first reduced from 81 in 2000 to 59 in 2015 and 56 in 2019 and then increased again to 59 in 2020. This increase is associated with the coronavirus disease 2019 (COVID-19) pandemic due to the disruption of malaria prevention plans. According to the WHO, the Southeast Asia region accounts for 2% of the global burden of malaria cases. India accounts for 83% of cases of malaria in this region [1].

Malaria has been a common cause of death in children below five years of age [2]. In 2020, the total death toll from malaria increased by 12% as compared with 2019 [3]. According to the WHO, the Southeast Asia region recorded 9,000 deaths due to malaria in 2020. India accounted for about 82% of all malaria deaths in this region [1]. Almost all the deaths occurring due to malaria are preventable and treatable.

There are a number of nationwide malaria prevention programs that have been implemented by the Government of India. The National Malaria Control Programme (NMCP) is one such program implemented in 1953, with a focus on indoor residual spraying of dichlorodiphenyltrichloroethane (DDT). The program helped to drastically reduce the incidence of malaria [4]. Following this program's success in reducing the burden of malaria, various government schemes and programs have come up over the years, which have helped to further reduce the burden of malaria in India.

In 2015, the prime minister of India endorsed a plan and roadmap to eliminate malaria throughout the region by 2030, alongside 17 East Asia Summit leaders [5]. The goal seems attainable now, as WHO has approved a vaccine for malaria called RTS,S/AS01 (RTS,S) [6]. It is the very first malaria vaccine to be approved to prevent malaria from *Plasmodium falciparum*. It is indicated for the active immunization of infants and children: three doses are to be given intramuscularly between five and 17 months, with a fourth booster dose at around age two.

Given the recent advancements in the field of malaria vaccine invention, it is imperative to understand the awareness and acceptance level of this vaccine in countries most susceptible to this life-threatening, preventable disease. This study set out with an aim of assessing the awareness and acceptance of the malaria vaccine among residents of India.

## Materials and methods

A cross-sectional study design was implemented across India. A questionnaire was developed by consensus of three investigators. It consisted of items on demographic characteristics, knowledge of malaria and its treatment and preventative measures, awareness of the malaria vaccine, and its acceptability for administration in children. The data were collected using Qualtrics® software (Provo, UT). The questionnaire was self-administered anonymously using a link shared with people across India through social media platforms like WhatsApp, Facebook, Instagram, Twitter, and Telegram. To participate in the study, respondents had to be 18 years and above and currently residing in India. The data were collected over a period of 12 weeks between December 2021 and March 2022. All the participants indicated consent before beginning the survey online.

Survey instrument

The questionnaire used for the survey included sociodemographic characteristics: age, residence, gender, education, and caregiver status. A survey item asked whether the respondent had heard about the malaria vaccine, an immunization used to help prevent malaria. The response options were “Yes” or “No.” Three items were included to assess acceptability. These included the following: “The malaria vaccine should be added to the required childhood immunization for all children”; “The malaria vaccine should only be recommended, parents/caregivers should decide if their children should receive the vaccine or not”; and “If the vaccine is readily available, and at no cost, I would encourage parent/guardians/caregivers to get their 0-5-year-old children vaccinated.” All three variables assessing attitude were assessed dichotomously (agree/disagree).

Data processing and analysis

The dataset originally had 2,637 observations. Observations with missing relevant data (n = 266) were excluded, bringing it down to 2,371 observations with data on the covariates of interest, which were then included in the analyses. The data were entered into and cleaned using SAS OnDemand for Academics (SAS Institute Inc., Cary, NC). Frequencies and proportions were first used to describe and summarize the data. The chi-square test was used to determine the associations between dependent and independent variables. A p-value of less than 0.05 was used to declare statistical significance.

## Results

The final sample included 2,371 participants from India. Out of which, 1032 were females. The majority of the respondents were in the age group of 18-24 years (37.8%); 41.8% were college-educated and 43.9% were living in large cities or metropolitans. Most respondents (62.76%) indicated that they were not caregivers to a child less than five years old. Table 1 shows the participants’ demographics.

**Table 1:** Sociodemographic characteristics of survey respondents

Characteristics		Frequency (%)
Age (years)	18-24	898 (37.87)
	25-34	863 (36.40)
	35-44	410 (17.29)
	45-54	125 (5.27)
	55-64	49 (2.07)
	65-74	21 (0.89)
	75-84	5 (0.21)
Gender	Female	1,032 (43.53)
	Male	1,321 (55.71)
	Prefer not to say	16 (0.67)
	Other	2 (0.08)
Education	No education	19 (0.80)
	Primary school	77 (3.25)
	Secondary school	550 (23.20)
	Undergraduate	992 (41.84)
	Graduate/professional	671 (28.30)
	Other	62 (2.61)
Residence	Hamlet (<100)	46 (1.94)
	Village (100-2,499	362 (15.27)
	Small town (2,500-9,999)	496 (17.97)
	Mid-sized town (10,000-49,999)	426 (17.97)
	Large city/metropolitan area (>50,000)	1,041 (43.91)
Caregiver status	Provides care to a child <5 years old	883 (37.24)
	Not a caregiver	1,488 (62.76)

Malaria vaccine awareness among participants

Out of all the participants who took the survey, 72% were aware of the malaria vaccine. Awareness was highest among the age group of 25-34 years, and age was statistically significantly associated with awareness (p < 0.0001). Males had higher knowledge of the vaccine as compared to the females in the country and gender (p = 0.017) showed a significant association with awareness. Knowledge of the vaccine was associated with higher education and being a caregiver to a child (p < 0.05). However, there was no relationship between awareness of the vaccine and their willingness to encourage others to take the vaccine (p > 0.05) (Table 2).

**Table 2:** Awareness of malaria by various demographic characteristics

Characteristics		Have not heard about the malaria vaccine	Have heard about the malaria vaccine	Sig (chi-square, p-value)
		Frequency (%)		
Age (years)	18-24	384 (16.20)	514 (21.68)	216.32, p < 0.0001
	25-34	145 (6.12)	718 (30.28)	
	35-44	54 (2.28)	356 (15.01)	
	45-54	48 (2.02)	77 (3.25)	
	55-64	21 (0.89)	28 (1.18)	
	65-74	12 (0.51)	9 (0.38)	
	75-84	1 (0.04)	4 (0.17)	
Gender	Female	305 (12.86)	727 (30.66)		9.67, p = 0.017
	Male	351 (14.80)	970 (40.91)		
	Prefer not to say	9 (0.38)	7 (0.30)		
	Other	0 (0.00)	2 (0.08)		
Education	No education	0 (0.00)	19 (0.80)	88.19, p < 0.0001	
	Primary school	2 (0.08)	75 (3.16)	
	Secondary school	182 (7.68)	368 (15.52)	
	Undergraduate	212 (8.94)	780 (32.90)	
	Graduate/professional	247 (10.42)	424 (17.88)	
	Other	22 (0.93)	40 (1.69)	
Residence	Hamlet (<100)	1 (0.04)	45 (1.90)		238.33, p < 0.0001
	Village (100-2,499	50 (2.11)	312 (13.16)		
	Small town (2,500-9,999)	58 (2.45)	438 (18.47)		
	Mid-sized town (10,000-49,999)	106 (4.47)	320 (13.50)		
	Large city/metropolitan area (>50,000)	450 (18.98)	591 (24.93)		
Caregiver status	Provides care to a child <5 years old	90 (3.80)	793 (33.45)	222.26, p < 0.0001	
	Not a caregiver	575 (24.25)	913 (38.51)	
Child vaccine required	No	53 (2.24)	76 (3.21)		11.49, p = 0.0007
	Yes	612 (25.81)	1,630 (68.75)		
Encourage others to take the vaccine	No	43 (1.81)	111 (4.68)	0.0013, p = 0.9715	
	Yes	622 (26.23)	1,595 (67.27)	
Vaccine should only be recommended	No	200 (8.44)	306 (12.91)		41.99, p < 0.0001
	Yes	465 (19.61)	1,400 (59.05)		
Total		665 (28.05)	1,706 (71.95)	

## Discussion

The availability and efficacy of the malaria vaccine RTS,S/AS01 have the potential to play a crucial role in controlling the spread of malaria. The vaccine in combination with current control measures has been shown to significantly reduce malaria infections [7,8]. To be effectively implemented, it is important that the public is aware of and accepting of the vaccine. The questionnaire was designed to assess awareness, acceptability, and general attitude toward the malaria vaccine.

To eradicate malaria, public knowledge, including awareness about disease transmission, preventative measures, and vaccine information, is important to promote vaccine acceptance and decrease vaccine hesitancy among the population [9]. The awareness of the malaria vaccine was high among the people of India (72%). However, the acceptability of the vaccine was not as high as awareness. Responses to the three items on vaccine acceptance were as follows: all children below five years old should be vaccinated (68.75%), encouraging others to get vaccinated (67.27%), and vaccines should only be recommended (59.05%). This discrepancy could be due to a lack of understanding regarding the safety of the vaccine or distrust. However, distrust in vaccine efficacy and safety varies in different sociodemographic groups as well as in vaccine availability [10,11].

Although India is currently designated as a low transmission setting and witnessed 77 malaria-related deaths in 2019, the rollout of the malaria vaccine is imminent. It will not only help India eliminate malaria by 2030 but also maintain the said status [12].

Previous studies have assessed the possible causes of vaccine hesitancy in India such as misinformation on social media, religious/traditional practices, cost of vaccines, and lack of awareness [13], Thus, government agencies, healthcare workers, and other stakeholders need to efficiently tackle the misinformation campaigns on social media and other peer communication platforms.

Several studies have assessed the awareness of the malaria vaccine, willingness to vaccinate, acceptance of the malaria vaccine, and other parameters in several African countries such as Nigeria [14,15], Tanzania [16], Ghana [17], and Ethiopia [18]. They have reported high awareness status and high prospects for pediatric immunization of malaria. The current study is novel in that it evaluated the awareness of the malaria vaccine in India. Moreover, previous studies that assessed malaria vaccine awareness were carried out before the WHO approved its usage. The current study provides primary evidence of vaccine awareness among the Indian populace after the recent approval by the WHO. There is a paucity of original studies [19] evaluating vaccine hesitancy and awareness status in India, which we have attempted to resolve via the present study. Although we have identified several studies on vaccine hesitancy for COVID-19 in India, due to its emergency use authorization, it might not be a good indicator of malaria and other vaccines’ hesitancy and awareness [19-21]. Further evaluation post introduction of the malaria vaccine by the Government of India is warranted, to calibrate the healthcare policy, especially in the face of the continuously changing healthcare scenario in India.

Limitations

The present study possessed a few limitations as the data collected were via an online survey across the country in English. Predictably, respondents enrolled in the study were educated and had access to social media platforms such as WhatsApp, Facebook, Twitter, Instagram, and LinkedIn, which could lead to response bias. Access to social media and the internet generally predisposes people to news such as the WHO's approval of the malaria vaccine. Additionally, only those participants who can read English responded to this survey. However, the latter case is an unavoidable conflict due to extensive linguistic diversity in India. The demographic distribution showed a higher percentage of males, age group of 18-24 years old, residing in large/metropolitan cities, educated, and are not care providers to children below five years old. Hence, the study population was not reflective of the target population that will make decisions on whether or not children should get the vaccine. Therefore, the results are not generalizable. This was a convenience sample and not representative of the Indian population. While not generalizable, it does provide information useful for further investigation and early policy formulation regarding any intended rollout.

## Conclusions

This paper assessed the public awareness of the malaria vaccine that was recently approved by the WHO. Findings show that males, participants between 18 and 24 years, and caregivers of children aged five years or less are more likely to be aware of the vaccine. Similarly, more educated respondents and those who reside in urban localities are more aware of the vaccine. As health policymakers develop immunization schemes for malaria, it is critical that they target groups that are less informed with relevant education and promotion efforts as the malaria vaccine has the potential to provide significant public health benefits to society.
